# Radically open dialectical behaviour therapy adapted for adolescents: a case series

**DOI:** 10.1186/s12888-021-03460-3

**Published:** 2021-09-22

**Authors:** Julian Baudinet, Catherine Stewart, Eleanor Bennett, Anna Konstantellou, Rhian Parham, Keren Smith, Katrina Hunt, Ivan Eisler, Mima Simic

**Affiliations:** 1 Maudsley Centre for Child and Adolescent Eating Disorders (MCCAED), Maudsley Hospital, De Crespigny Park, Denmark Hill, London, SE5 8AZ UK; 2 Institute of Psychiatry, Psychology & Neuroscience (IoPPN), King’s College London, De Crespigny Park, Denmark Hill, London, SE5 8AF UK; 3 National and Specialist Child and Adolescent Dialectical Behaviour Therapy Service (N&S CAMHS DBT), Maudsley Hospital, De Crespigny Park, Denmark Hill, London, SE5 8AZ UK

**Keywords:** Adolescent_1_, Eating disorders_2_, Self-harm_3_, Depression_4_, Radically open dialectical behaviour Therapy_5_, Dialectical behaviour Therapy_6_, Overcontrol_7_

## Abstract

**Background:**

Overcontrol is a transdiagnostic cluster of traits associated with excessive psychological, behavioural and social inhibitory control. It is associated with psychiatric diagnoses of depression, restrictive eating disorders and/or obsessive-compulsive personality disorder. Radically Open Dialectical Behaviour Therapy is a transdiagnostic treatment for maladaptive overcontrol. This case series evaluates an adolescent adaption (RO-A) for a transdiagnostic group of adolescents identified as overcontrolled.

**Methods:**

Twenty-eight adolescents were consecutively referred for RO-A from two different National and Specialist Child and Adolescent Mental Health Services between June 2017 and February 2020. Baseline self-report measures assessed overcontrol characteristics, relationship and attachment quality and mental health symptoms of depression and eating disorders, which were repeated at discharge.

**Results:**

Adolescents in this case series reported high rates of depression (78.6%), self-harm (64.3%) and eating disorders (78.6%). Most (85.7%) had two or more mental health diagnoses and all had previous mental health treatments before starting RO-A. The mean number of RO-A sessions attended was 18 group-based skills classes and 21 individual sessions over a mean period of 34 weeks. Significant improvements with medium and large effect sizes were reported in cognitive flexibility (*d* = 1.63), risk aversion (*d* = 1.17), increased reward processing (*d* = .79) and reduced suppression of emotional expression (*d* = .72). Adolescents also reported feeling less socially withdrawn (*d* = .97), more connected to others (*d* = 1.03), as well as more confident (*d* = 1.10) and comfortable (*d* = .85) in attachment relationships. Symptoms of depression (*d* = .71), eating disorders (*d* = 1.06) and rates of self-harm (*V* = .39) also significantly improved. Exploratory correlation analyses suggest improvements in overcontrol are moderately to strongly correlated with improvements in symptoms of depression and eating disorders.

**Conclusions:**

This case series provides preliminary data that RO-A may be an effective new treatment for adolescents with overcontrol and moderate to severe mental health disorders like depression and eating disorders. RO-A led to improved management of overcontrol, improved relationship quality and reduced mental health symptoms. Further evaluation is indicated by this case series, particularly for underweight young people with eating disorders. More rigorous testing of the model is required as conclusions are only tentative due to the small sample size and methodological limitations.

## Background

Overcontrol is a transdiagnostic cluster of characteristics associated with excessive inhibitory control [1]. This cluster of interrelated characteristics includes: cognitive and behavioural inflexibility, supressed emotional expression, perfectionism, heightened performance monitoring, increased threat sensitivity, and reduced reward processing [1, 2]. This is typically coupled with a reduced sense of social connection and increased isolation irrespective of the size of one’s social circle or frequency of social contact [1, 3, 4]. Overcontrol has been associated with a range of psychiatric diagnoses, including refractory depression [1, 4], restrictive eating disorders [3, 5] and obsessive-compulsive personality disorder [1], as well as paediatric anxiety disorders [2].

Overcontrol is hypothesised to result from the interaction of neurobiological, environmental and learning factors [1]. It can be expressed discreetly, and difficulties are not always overtly obvious when interacting with others. People with this cluster of traits describe experiencing high levels of negative emotions, whilst displaying an outwardly inhibited or sometimes overly agreeable facade. This can make overcontrol difficult to identify and target in treatments [1]. While psychological treatments are not expected to result in temperamental change, typically considered neurobiological and genetically based [6], new treatments can aim to support individuals to understand, identify and manage temperamental factors in more adaptive ways. Given the high rates of comorbidity [7–9], relapse [10–13], and treatment non-response [9, 14, 15] for individuals with the aforementioned cluster of diagnoses, treatments that target underlying transdiagnostic mechanisms and reconceptualise treatment targets to the management of broader temperamental and personality factors may help to improve outcomes and reduce relapse rates.

Radically Open Dialectical Behaviour Therapy (RO DBT) is a new transdiagnostic treatment that targets maladaptive overcontrol [3]. It is provided over approximately 8 months and consists of a combination of weekly skills classes (groups) and weekly individual sessions. Treatment primarily focuses on improving social connection via the change mechanisms of a) reducing physical arousal associated with threat sensitivity, b) more open and genuine emotional expression and c) improved social signalling [1, 16]. Social signalling refers to the intended and unintended cues people constantly display to others. RO DBT posits that improved social signalling leads to the development of closer and more genuine social connections, which then leads to improved symptom management and reduction of psychological distress. There is now evidence that RO DBT is effective for treating refractory depression [4, 17, 18] and preliminary evidence for the treatment of adult eating disorders [19–21]. Despite these promising findings with adults, RO DBT is yet to be empirically tested with adolescents beyond its use in a day program setting for adolescents with eating disorders [22].

RO DBT was introduced as a new transdiagnostic treatment in partnership between two National and Specialist Child and Adolescent Mental Health Services (N&S CAMHS) services; the Maudsley Centre for Child and Adolescent Eating Disorders (MCCAED) and the Dialectical Behaviour Therapy (DBT) Service at the Maudsley Hospital in London. RO DBT was piloted in its original form from 2015 to 2016. Feedback from adolescents who received RO DBT during this early pilot testing period resulted in modifications of the original RO DBT materials to make them more developmentally sensitive and appropriate for an adolescent population. Structurally the treatment was shortened from 30 (120 min) down to 20 (90 min) weekly skills classes provided alongside weekly individual sessions (60 min). Some of the original RO DBT skills were simplified, combined and/or the language was changed to be more adolescent appropriate. Similarly, examples in the RO DBT materials were modified to be more relevant and relatable to this age group. Lastly, images, video clips and new activities were introduced to improve engagement with the materials and concepts. The structure and timing of the weekly individual sessions remained unchanged.

This case series aimed to assess whether the adolescent adaptation of Radically Open Dialectical Behaviour Therapy (RO-A) leads to improvements in overcontrol characteristics, relationship quality, and psychiatric symptoms of depression and eating disorders. The study also explored whether any changes were consistent with the theoretical model of change proposed by the RO DBT treatment model that improvements in overcontrol are associated with improvements in psychiatric symptoms. This article reports the findings for the initial phase of evaluation of RO-A.

## Method

### Participants

Adolescents (13–18 years old at baseline) in this study were referred from either the DBT service or MCCAED at the Maudsley Hospital. This case series reports on consecutive referrals between June 2017 and February 2020, the period in which the new adolescent adapted RO-A treatment programme was being delivered. All adolescents were screened for overcontrolled personality traits using the Assessing Styles of Coping Word-pair Checklist (ASC-WP) [1] followed by clinical interview assessing overcontrol factors such as risk aversion, perfectionism, emotional expressiveness, social connectedness, and rigid and rule governed behaviour.

Adolescents referred from the DBT service were all initially referred to standard DBT for treatment of repeated episodes of self-harm and low mood. If, during the initial assessment with the service, overcontrol was identified using the ASW-WP screening tool and clinical interview, RO-A was offered rather than standard DBT.

All adolescents referred from MCCAED were screened for overcontrolled using the same procedure (ASC-WP screen and clinical interview) if, after receiving family therapy for eating disorder (FT-ED), they continued to experience high levels of eating disorder behaviours and cognitions that interfered with daily functioning despite partial or full weight restoration. Persisting difficulties included, ongoing significant distress at mealtimes, significant cognitive rigidity and rules around food and eating, and/or significant social and education disruption due to these factors (e.g., missing school, struggling to socialise).

**Fig. 1:**
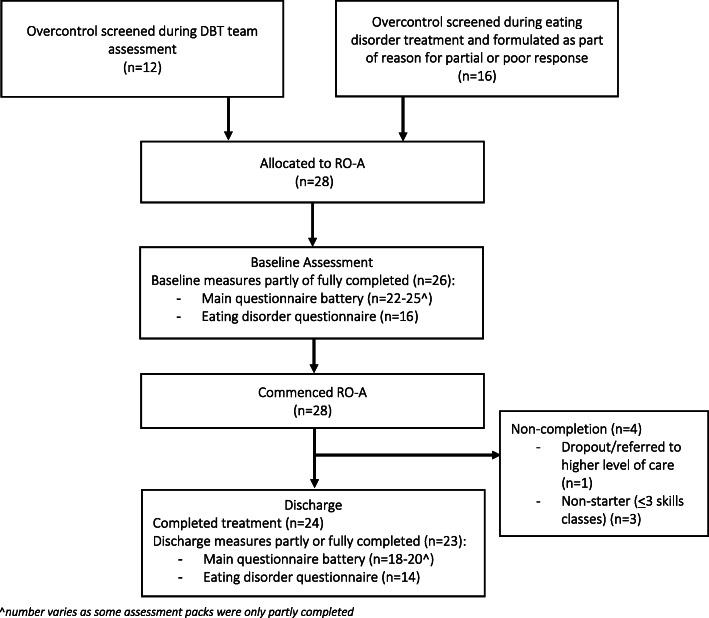
Participant Flowchart

Exclusion criteria for this RO-A case series included psychosis, medical instability (see Junior MARSIPAN guidelines [23]), high psychiatric risk requiring inpatient treatment (e.g. imminent suicidal risk), emotional undercontrol and/or previous experience of RO DBT. No minimum weight was required for inclusion. See Fig. 1 for study flowchart.

### Treatment intervention and model

Treatment in this study is an adolescent adaptation (RO-A) of the original RO DBT model described by Lynch [16]. See above for more details on the modifications made. These changes were based on early feedback from adolescents that treatment length was too long and that materials were too adult focused.

RO-A includes 20 weekly 90-min skills class and a weekly 60-min individual session. Skills classes focus on teaching new skills to manage maladaptive overcontrol and includes mindfulness practice, homework provision and review. Skills classes consist of between two to eight individuals in treatment working together with one or two facilitators depending on the group size. The skills class focusses on teaching a range of skills designed to help adolescents express emotions more freely, engage in new novel behaviours, increase spontaneity and playfulness, live more flexibly, learn from feedback, strengthen social and community connectedness, and activate social safety systems. Individual sessions focus on applying these skills in the adolescent’s daily life, monitoring social signalling and overt overcontrolled behaviours, linking these with internal experiences and value-based goals. This includes the use of diary cards, in-session role plays and the use of chain analyses. See treatment manual for further details of treatment aims and structure [1, 16].

All adolescents were initially contracted to attend one full round of skills classes (*n* = 20) after which treatment was reviewed. Actual treatment length was based on individual goals and symptom presentation. Once adolescents had reached their identified value-based goals treatment ended, regardless of the number of individual or skills classes they had attended. Additional individual sessions and/or skills classes was offered if adolescents were actively working towards their value-based goals and using treatment effectively.

### Treatment objectives

RO-A aims to reduce maladaptive overcontrol by targeting emotional expressiveness, cognitive flexibility, and social signalling. Improved social functioning and social signalling is hypothesised to lead to improved social connection, psychiatric symptom improvement and more global improvements in functioning.

### Therapists

All therapists involved in this study were employed by either the N&S CAMHS DBT service or MCCAED. RO DBT therapists represented the mix of professions present in both multidisciplinary teams, including clinical and counselling psychology, psychiatry, family therapy and nursing. All therapists attended 10-days of intensive RO DBT training delivered by approved RO DBT trainers, and attended weekly to fortnightly RO DBT consult with bi-monthly external supervision by a RO DBT approved supervisor.

### Ethics approval and consent to participate

This study was approved by the South London and Maudsley (SLaM) CAMHS Service Evaluation and Audit Committee. As this study constitutes service evaluation or audit, NHS Research Ethics Committee approval was not required. SLaM CAMHS service evaluation and audit approval allows for analysis and publication of anonymised data extracted from case files without written consent from participants or carers. Outcome measures were administered as part of routine clinical care. All methods were performed in accordance with the stipulated guidelines and regulations.

### Data collection and outcome measures

Outcome in this case series was measured as changes in overcontrol characteristics, relationship quality and psychiatric symptoms of depression and eating disorders. A range of self-report questionnaires were included that were selected to identify temperament, personality and coping factors associated with overcontrol in adolescents, as well as relationship quality and attachment. Validated adolescent measures were not available for the full range of overcontrol related factors as this is an emerging field. Adult measures were used in their absence. Symptoms of depression were also assessed using self-report measures to explore the relationship between changes in overcontrol factors and changes in psychiatric symptoms. Eating disorder symptoms were also assessed for those who reported eating concerns at assessment. Outcome measures were collected by clinical staff as part of routine clinical care.

#### Measures for screening and assessing overcontrol characteristics

The Assessing Styles of Coping Word-pair Checklist (ASC-WP) [1] was used as the initial screen for overcontrol. This 47-item self-report screening tool requires participants to choose one word from a pair of words that best describes them. Word pairs include one word that is more representative of over- and the other of undercontrol. The ASC-WP has not been validated with young people but was included due to an absence of any validated screening tools for overcontrol at the time of data collection.

The Emotion Regulation Questionnaire (ERQ) [24] is a validated 10-item self-report measure used to examine emotional regulation strategies via two subscales: cognitive reappraisal and the suppression of emotional expression. Cognitive reappraisal strategies refer to when someone changes their cognitions in order to change their emotional experience (example item: “when I want to feel less negative emotions [such as sadness or anger], I change what I’m thinking about”). The expressive suppression subscale assesses how much someone inhibits the behavioural expression of their emotions to regulate themselves (example item: “when I am feeling negative emotions, I make sure not to express them”). Cognitive reappraisal strategies are typically considered adaptive and associated with low psychological distress, whereas expressive suppression is considered less adaptive and associated with psychological distress and alexithymia [25]. The ERQ has demonstrated good reliability and validity [24], good internal consistency [25], and has been used with adolescents [26]. Internal consistency in the current study was good for the Reappraisal subscale (baseline a = .92; discharge a = .88), and moderate for the Suppression subscale (baseline a = .77; discharge a = .79).

The Negative Temperament subscale of the Schedule of Non-adaptive and Adaptive Personality for Youth (SNAPY-Y) [27] was included to assess level of maladaptive negative temperament and its stability across treatment. The subscale measures tendencies towards irritability, distress, fear, anger and sadness. The SNAP-Y has shown to be a valid measure of personality in adolescence that demonstrates good internal consistency, structural validity [27], and has available clinical norms [27, 28]. Internal consistency was moderate to good in the current study (baseline a = .78, discharge a = .81).

The Five Factor Obsessive Compulsive Inventory – Short Form (FFOCI) [29, 30] is a 48-item self-report assessment of risk aversion, cognitive flexibility, perfectionism, workaholism and punctiliousness. The FFOCI has not been validated for children and adolescents, but in the absence of a validated measure of obsessive-compulsive personality traits in children and adolescence, was included in this study. The FFOCI has demonstrated good discriminant validity and internal consistency with an undergraduate university sample [30]. Internal consistency was variable in the current study and ranged from good to poor depending on the subscale (Risk Aversion baseline a = .68, discharge a = .61; Inflexibility baseline a = .59, discharge a = .81; Punctiliousness baseline a = .77, discharge a = .72; Perfectionism baseline a = .60, discharge a = .74; Workaholism baseline a = .86, discharge a = .89).

Reward processing was assessed using the Temporal Experience of Pleasure (TEPS) [31]. The 18-item self-report measure assesses two aspects of trait-based reward processing based on Klein’s [32] model of anhedonia. Anticipatory pleasure (TEPS-ANT; “wanting”), the first subscale, examines the motivation for and expectation of pleasure and reward responsivity. The second subscale, consummatory pleasure (TEPS-CON; “liking”), measures the appreciation of positive stimuli and openness to different experience in the moment. Anticipatory, as opposed to the consummatory, aspects of reward processing have been associated with motivation, reinforcement learning and reward-based decision-making [33]. The TEPS has not been validated with adolescents but has demonstrated good convergent and divergent validity, internal consistency and test-retest reliability in undergraduate university samples [31]. Internal consistency within the current study was moderate to good (TEPS-ANT baseline a = .90, discharge a = .82; TEPS-CON baseline a = .69, discharge a = .84).

#### Measures assessing relationships quality

The Withdrawal subscale of the Youth Self-Report questionnaire (YSR-W) [34] is an 8-item self-report measure examining the degree of perceived social withdrawal and isolation. The YSR is a valid, reliable and frequently used measure to assess a range of problems in adolescents [34]. Internal consistency was moderate to good in the current study (baseline a = .79, discharge a = .86).

The Social Connectedness Scale (SCS-R) [35] is a 20-item self-report measure used to assess connectedness that an individual feels in their social environment. Low scores are indicative of low levels of social connection. This measure shows good internal consistency and validity with an adult sample [35], however has not been validated with adolescents. Internal consistency was high in the current study (baseline a = .92, discharge a = .91).

The Attachment Styles Questionnaire (ASQ) [36] was used to define attachment characteristics and the quality of parental relationships. The ASQ consists of 40-items partitioned into five subscales including relationship confidence, need for approval, discomfort with closeness, pre-occupation and relationships as secondary. The ASQ has been shown to be valid and reliable, with good internal consistency [36–38] and has been used with adults and adolescents [39]. Internal consistency ranged from good to poor in the current study, depending on the subscale (Confidence baseline a = .79, discharge a = .83; Discomfort baseline a = .86, discharge a = .84; Preoccupation baseline a = .70, discharge a = .70; Relationships as Secondary baseline a = .75, discharge a = .59; Need for Approval baseline a = .77, discharge a = .73).

#### Diagnostic assessment and measures of mental health symptoms

All adolescents in this case series completed the Development and Wellbeing Assessment (DAWBA) at assessment. The DAWBA is a widely used structured diagnostic assessment that generates DSM-5 [40] and ICD-10 [41] psychiatric diagnoses for two to 17-year olds [42]. It has been shown to be a valid diagnostic tool [43] and may be more suitable than the widely used Eating Disorder Examination (EDE) diagnostic interview [44] for diagnosing adolescents with an eating disorder [45].

The Moods and Feelings Questionnaire (MFQ) [46] consists of 33-items used to screen for symptoms of depression in children and young adults. Scores of 27 and higher indicate the presence of depression [47, 48]. The MFQ was provided to all adolescents at baseline and discharge, regardless of symptom presentation. It has been shown to have good validity, reliability and internal consistency with adolescents [49]. Internal consistency was good in the current study (baseline a = .92, discharge a = .91).

Incidence of self-harm was collected at baseline and discharge using a single-item questions. Adolescents self-reported whether or not they had engaged in any self-harm in the preceding 2 weeks.

The Eating Disorder Examination Questionnaire (EDE-Q, v6) was completed at baseline and discharge by those who reported eating concerns at assessment (*n* = 23). The EDE-Q is a 28-item measure with a total (global) score made up by four subscales: restraint, eating concerns, shape concerns and weight concerns. It has good internal consistency [50] and has been used previously with clinical [51] and community adolescent samples [52]. Internal consistency was moderate to good, depending on the subscale (Global Score baseline a = .79, discharge a = .83; Dietary Restraint baseline a = .86, discharge a = .84; Eating Concerns baseline a = .70, discharge a = .70; Shape Concerns baseline a = .75, discharge a = .59; Weight Concerns baseline a = .77, discharge a = .73).

Percentage of median Body Mass Index (%mBMI) adjusting for age and gender (BMI/median BMI for age and gender × 100) was also recorded to assess changes in physical health for those who reported eating concerns. This is the recommended method for children and adolescents with anorexia nervosa [23]. In this study, any young person under 90%mBMI was classified as underweight, and under 85%mBMI as significantly underweight.

### Statistical analysis

The Shapiro-Wilkes test was used to test the distribution of the data. Paired t-tests were used for normally distributed data and Wilcoxon signed-rank test for non-normally distributed data to compare differences between baseline and discharge data. Cohen’s d was used to measure effect sizes for the paired t-tests (> 0.3 = small; > 0.5 = medium; > 0.7 = large). Non-parametric data effect size was estimated using *r* (> 0.1 = small; > 0.3 = medium, > 0.5 = large). McNamar’s test was used to compare rates of self-harm (present/absent) in the 2 weeks preceding assessment and the 2 weeks preceding discharge. Effect size was estimated using Cramer’s V. Internal consistency for each measure and subscale was assessed using Cronbach’s alpha. Pearson’s r correlations were conducted to explore the relationship between changes in overcontrol related factors and changes in symptoms of depression and eating disorders from baseline to discharge. Due to the exploratory nature and sample size, significance testing was not conducted, rather 95% confidence intervals are reported. All statistical analyses were performed using SPSS version 26.

To examine potential sampling bias in missing data at discharge, analyses were conducted to compare those who had paired data (completed assessment measures at both baseline and discharge) to those who did not across key demographic and clinical factors. Results showed that there were no differences between those with paired data compared to those without with regard to age, referral team (MCCAED or DBT service), primary diagnostic category (eating, mood or anxiety disorder diagnosis), severity of mood symptoms (MFQ at baseline) or the presence of self-harm. For the subgroup referred with eating concerns there was also no difference in weight (%mBMI) or severity of eating disorder psychopathology (EDEQ Global score) at baseline. Further analysis was conducted to examine difference in treatment characteristics. There was no difference between those with paired data and those without with regard to duration of treatment (in weeks), the number of skills classes attended, or the number of individual sessions attended.

## Results

### Group characteristics

Twenty-eight adolescents who met the case series inclusion criteria were offered RO-A between June 2017 and February 2020. Sixteen (57.1%) were referred from MCCAED and 12 (42.9%) from DBT. Demographic information is presented in Table 1. The majority were female (92.9%) and identified as White British (71.4%). One identified as transgender. Rates of major depressive disorder and eating disorders were both high (MDD = 78.6%; ED = 78.6%) for the group as a whole. Twenty-three adolescents reported eating concerns at assessment. Of these, 22 met DSM-5 criteria for an eating disorder diagnosis. All but one (*n* = 21/22, 95.2%) had an eating disorder primarily characterised by restrictive eating (anorexia nervosa and atypical anorexia nervosa). Mean weight at the start of RO-A for the young people diagnosed with an eating disorder was 94.65%mBMI (sd = 6.63, range = 83.20–109.00). Three young people were underweight (< 90%mBMI), and one was significantly underweight (< 85%mBMI).

**Table 1:** Patient characteristics (*N* = 28)

Age range in years (mean)	13–18 (16.1)
Gender distribution (%)	26 females (92.9%), 2 males (7.1%)
Ethnicity	
- White British	20 (71.4%)
- Black British	1 (3.6%)
- British Indian	2 (7.1%)
- Other	5 (17.9%)
DSM-V Diagnoses	
- Eating disorder	22 (78.6%)
- *Anorexia nervosa (AN)*	*12 (42.9%)*
- *Atypical anorexia nervosa (AN-A)*	*9 (32.1%)*
- *Bulimia nervosa (BN)*	*1 (3.6%)*
- Major Depressive Disorder	22 (78.6%)
- Anxiety disorder (> 1 diagnosed)	19 (67.9%)
- *Generalised Anxiety Disorder*	*17 (60.7%)*
- *Social Phobia*	*12 (42.9%)*
- *Separation Anxiety*	*1 (3.6%)*
- *PTSD*	*1 (3.6%)*
- *Panic Disorder*	*2 (7.1%)*
- *Specific Phobia*	*2 (7.1%)*
→ *Agoraphobia*	*1 (3.6%)*
- OCD	2 (7.1%)
- Autism Spectrum Disorder	1 (3.6%)
Number of diagnoses	
- 1 diagnosis	4 (14.3%)
- 2 diagnoses	8 (28.6%)
- 3 diagnoses	8 (28.6%)
- 4 or more diagnoses	8 (28.6%)
Previous treatment	
- FT-ED	20 (71.4%)
- Systemic family therapy	1 (3.6)
- CBT	13 (46.4%)
- DBT	1 (3.6%)
- Inpatient treatment	8 (28.6%)
- 2 or more previous treatments	7 (25.0%)
- No previous treatment	0 (0%)

*Abbreviations*: *PTSD* Post-traumatic stress disorder, *OCD* Obsessive-compulsive disorder, *OSFED-R* Other specified feeding and eating disorders characterised by restriction, *FT-ED* Eating disorder focused family therapy, *DBT* Dialectical Behaviour Therapy, *CBT* Cognitive Behavioural Therapy

Comorbidity was the norm, with 85.7% meeting criteria for two or more DSM-5 psychiatric diagnoses. All adolescents (100.0%) had received at least one type of psychological treatment prior to attending RO-A. Fourteen young people (2/16 MCCAED referrals, 12/12 DBT referrals) had engaged in treatment with general CAMHS prior to RO-A. Mean duration of CAMHS treatment was 22.71 months (sd = 15.23, range = 3–54 months), and mostly consisted of cognitive behaviour therapy (CBT). For those referred to RO-A from MCCAED, the mean duration of treatment was 9.94 months (sd = 6.49, range = 3–29). All had received eating disorder focussed family therapy (FT-ED) and four had also received adjunctive CBT. See Table 1 for further details.

### Treatment characteristics

Twenty-four (85.7%) completed RO-A treatment, defined as a) reaching their value-based goals agreed at assessment and b) agreement between the young person and team about readiness. The mean number of skills classes attended was 18.42 (sd = 6.40, range = 9–34) and individual sessions was 20.82 (sd = 8.27, range = 8–42). Six young people (21.4%) attended more than 20 skills classes (one complete round), and two (7.1%) attended more than 25 skills classes. Attendance at skills class was high (mean DNA rate = 1.79, sd = 2.08, range = 0–7, median = 1.5). The mean treatment duration was 34.26 weeks (sd = 11.04, range = 15–62). Four people (14.3%) were identified as treatment non-completers. Of these, three attended three or fewer skills classes, and one dropped out of treatment after 10 skills classes and nine individual sessions due to weight loss and was referred to more intensive day programme treatment.

### Outcomes in Overcontrol characteristics

Descriptive and inferential statistics for characteristics of overcontrol are presented in Table 2. Suppression of emotional expression (ERQ-Suppression, *d* = −.72), cognitive inflexibility (FFOCI-inflexibility, *d* = 1.63) and the anticipatory aspects of reward processing (TEPS-ANT, *d* = .79) improved significantly from baseline to discharge with large effect sizes. Temperamental tendencies towards irritability, distress, fear, anger and sadness (negative temperament) did not significantly change from baseline to discharge (SNAP-Y Neg. Temp, *d* = .22).

**Table 2:** Overcontrol characteristics at baseline and discharge

Outcome measure (n paired)	Baseline	Discharge	Sig	Effect size
*Parametric*	*Mean (SD)*	*Mean (SD)*		
ERQ-Reappraisal (*n* = 14)	17.67 (8.60)	17.80 (6.92)	*p =* .96	*d* = .01
ERQ-Suppression (*n* = 15)	20.69 (4.00)	17.19 (5.17)	*p =* .01*	*d* = .72
TEPS-ANT (*n* = 16)	24.59 (10.82)	32.71 (9.80)	p = .005**	*d* = .79
TEPS-CON (*n* = 17)	29.78 (7.34)	31.00 (9.02)	*p* = .50	*d* = .16
FFOCI-Risk Aversion (*n* = 16)	14.47 (2.83)	11.35 (2.57)	*p* < .001**	*d* = 1.17
FFOCI-Inflexibility (*n* = 16)	13.82 (2.83)	10.65 (3.06)	*p* < .001**	*d* = 1.63
FFOCI-Punctiliousness (*n* = 15)	14.25 (2.90)	13.44 (3.61)	*P* = .35	*d* = .24
SNAP-Y-Neg. Temp. (*n* = 16)	33.76 (3.82)	34.71 (4.82)	*P* = .38	*d* = .22
*Non-parametric*	*Median (IQR)*	*Median (IQR)*		
FFOCI-Perfectionism (*n* = 17)	16 (13.5–16)	16 (14–16)	*p* = .26	*r =* .19
FFOCI-Workaholism (*n* = 16)	14 (12.25–14)	12.5 (7.25–12.5)	*p* = .14	*r-*.26

* = significant at *p* < 0.05 level; ** = significance at *p* < 0.01 level

*Abbreviations*: *ERQ* Emotion Regulation Questionnaire, *TEPS-ANT* Temporal Experience of Pleasure Scale-Anticipatory subscale, *TEPS-CON* Temporal Experience of Pleasure Scale-Consummatory subscale, *FFOCI-SF* Five Factor Obsessive Compulsive Inventory-Short Form, *SNAP-Y* Schedule for Nonadaptive and Adaptive Personality for Youth

Descriptive and inferential statistics for measures assessing relationship quality are presented in Table 3. There was a significant increase in social connectedness (SCS-R, *d =* 1.03) and significant reduction in perceived withdrawal (YSR-W, *d* = .97) from baseline to discharge, both with large effect size. Within attachment relationships, confidence (ASQ-Confidence, *d* = 1.10) significantly increased, whereas discomfort (ASQ-Discomfort, *d* = .85) and avoidance (ASQ-Relationships as Secondary, *d* = 1.14) significantly reduced from baseline to discharge, all with large effect size. The need for approval and preoccupation within attachment relationships did not significantly change.

**Table 3:** Relationship quality and attachment at baseline and discharge

Outcome measure (n paired)	Baseline *Mean (SD)*	Discharge *Mean (SD)*	Sig	Effect size
SCS-R (*n* = 16)	50.65 (11.51)	68.00 (15.05)	*p =* .001**	*d* = 1.03
YSR-W (*n* = 17)	12.17 (3.15)	8.50 (3.60)	*P* = .001**	*d* = .97
ASQ-Confidence (*n* = 17)	16.06 (4.78)	22.17 (6.36)	*p* < .001**	*d* = 1.10
ASQ-Discomfort (*n* = 17)	52.28 (5.77)	48.06 (6.92)	*p =* .002**	*d* = .85
ASQ-Preoccupation (*n* = 17)	33.61 (5.26)	35.78 (5.63)	*p* = .17	*d* = .34
ASQ-Relat. as Second. (*n* = 16)	23.18 (5.32)	16.82 (4.19)	*p* < .001**	*d* = 1.14
ASQ-Need for Approval (*n* = 17)	36.67 (4.34)	35.39 (4.05)	*p* = .21	*d* = .31

* = significant at *p* < 0.05 level; ** = significance at *p* < 0.01 level

*Abbreviations*: *SCS-R* Social Connectedness Scale-Revised, *YSR-W* Youth Self Report-Withdrawal subscale, *ASQ* Attachment Style Questionnaire

### Outcomes in psychiatric symptoms

Symptoms of depression (MFQ) reduced significantly from baseline (mean = 47.47, sd = 11.4) to discharge (mean = 36.76, sd = 17.38; *p* = .03) with large effect size (*d* = .71). Those with eating disorder concerns at baseline reported a significant reduction in eating disorder symptoms (EDE-Q Global Score) with large effect size (mean_baseline_ = 3.8, sd = 1.55; mean_discharge_ = 2.64, sd = 1.48, *p* = .04, *d* = 1.06). A significant reduction was also observed on all subscales of the EDE-Q; namely Dietary Restraint (mean_baseline_ = 3.42, sd = 1.84; mean_discharge_ = 1.86, sd = 1.32, *p* = .006, *d* = 1.13), Eating Concerns (mean_baseline_ = 3.76, sd = 1.42; mean_discharge_ = 2.60, sd = 1.60, *p* = .02, *d* = .86), Shape Concerns (mean_baseline_ = 4.64, sd = 1.64; mean_discharge_ = 3.52, sd = 1.88, *p* = .04, *d* = .75), and Weight Concerns (mean_baseline_ = 4.04, sd = 1.63; mean_discharge_ = 2.74, sd = 1.93, *p* = .04, *d* = .78).

Weight did not significantly change between baseline (mean = 94.65%mBMI, sd = 6.63, range = 83.20–109.00) and discharge (mean = 96.90%mBMI, sd = 7.86, range = 83.00–109.00, *p* = .21). Exploration of paired data revealed that one adolescent who was underweight at baseline lost weight (treatment non-completer), whereas the other two who were underweight at assessment were above 90%mBMI at discharge. All others maintained or slightly gained weight during RO-A.

Incidence of self-harm significantly reduced from baseline to discharge (*p* = .001, *V* = .39). Eighteen (64.3%) participants reported self-harm at assessment. Of these, five continued to report self-harm at discharge. No participant commenced self-harm during treatment.

### Correlation analysis

See Table 4 for exploratory correlation analysis exploring the relationship between changes in overcontrol factors and changes in symptoms of depression and eating disorders. A reduction in symptoms of depression was strongly correlated with changes in the anticipatory aspects of reward processing (TEPS-ANT, *r* = −.66). Medium correlations were observed between changes in depression symptoms and changes in the suppression of emotional expression (ERQ-Suppression, *r* = .43) and cognitive reappraisal (ERQ-Reappraisal, *r =* −.45) aspects of emotion regulation. Changes in depressive symptoms were only weakly correlated with changes in level of risk aversion (FFOCI-Risk Aversion, *r =* .23). A reduction in symptoms of depression correlated strongly with increased social connectedness (SCS-R, *r* = −.57) and reduced social withdrawal (YSR-W, *r* = .69). Within attachment relationships, a reduction in symptoms of depression from baseline to discharge was strongly correlated with increased confidence (ASQ-Confidence, *r* = −.49), reduced discomfort (ASQ-Discomfort, *r* = .74), reduced preoccupation (ASQ-Preoccupation, *r* = .48) and reduced need for approval (ASQ-Need for approval, *r* = .68).

**Table 4:** Correlation analysis examining the relationship between changes in overcontrol factors and changes in symptoms of depression and eating disorders

	Δ Depression Symptoms (MFQ)	Δ Eating Disorder Symptoms (EDE-Q Global)
Overcontrolled characteristics (*r*)		
Δ ERQ-Reappraisal	−.45 (CI: −.80, .14)	−.52 (CI: −.90, .30)
Δ ERQ-Suppression	.43 (CI: <−.01, .83)	.43 (CI: −.33, .85)
Δ TEPS-ANT	−.66 (CI: −.88, −.19)	−.41 (CI: −.84, .35)
Δ TEPS-CON	.49 (CI: −.80, .03)	−.40 (CI: −.82, .31)
Δ FFOCI-Risk aversion	.23 (CI: −.50, .68)	.09 (CI: −.61, .71)
Δ FFOCI-Inflexibility	.04 (CI: −.56, .50)	.27 (CI: −.48, .79)
Δ FFOCI-Perfectionism	−.56 (CI: −.84, −.04)	−.53 (CI: −.88, .21)
Δ FFOCI-Workaholism	−.34 (CI: −.75, .26)	−.45 (CI: −.88, .38)
Δ FFOCI-Punctiliousness	−.57 (CI: −.86, −.04)	−.53 (CI: −.88, .21)
Relationship quality and attachment (r)		
Δ SCS-R	−.57 (CI: −.85, −.06)	−.41 (CI: −.85, .35)
Δ YSR-W	.69 (CI: .28, .89)	.34 (CI: −.40, .79)
Δ ASQ-Confidence	−49 (CI: −.80, .03)	−.40 (CI: −.82, .31)
Δ ASQ-Discomfort	.74 (CI: .37, .91)	.57 (CI: −.09, .88)
Δ ASQ-Preoccupation	.48 (CI: −.05, .80)	.01 (CI: −.63, .63)
Δ ASQ-Relat. as second.	.16 (CI: −.41, .64)	<−.01 (CI: −.67, .66)
Δ ASQ Need for approval	.68 (CI: .26, .89)	.39 (CI: −.32, .82)

Δ = change (discharge score minus baseline score); CI = 95% confidence interval

*Abbreviations*: *ERQ* Emotion Regulation Questionnaire, *TEPS-ANT* Temporal Experience of Pleasure Scale-Anticipatory subscale, *TEPS-CON* Temporal Experience of Pleasure Scale-Consummatory subscale, *FFOCI-SF* Five Factor Obsessive Compulsive Inventory-Short Form, *SNAP-Y* Schedule for Nonadaptive and Adaptive Personality for Youth, *SCS-R* Social Connectedness Scale-Revised, *YSR-W* Youth Self Report-Withdrawal subscale, *ASQ* Attachment Style Questionnaire, *MFQ* Mood and Feelings Questionnaire, *EDEQ* Eating Disorder Examination Questionnaire

For the eating disorders subgroup, a reduction in eating disorder symptoms was strongly correlated with an increase in the use of cognitive reappraisal strategies to regulate emotions (ERQ-Reappraisal, *r* = −.52). It was moderately associated with improvements in both anticipatory (TEPS-ANT, *r* = −.41) and consummatory (TEPS-CON, *r* = −.40) aspects of reward processing, as well as flexibility (FFOCI-Inflexibility, *r* = .27), and the supression of emotional expression (ERQ-Supression, r = .43).

Eating disorder symptom reduction was also moderately correlated with improved social connectedness (SCS-R, *r* = − 41) and perceived social withdrawal (YSR-W, *r* = .34). Eating disorder symptom reduction was also strongly correlated with reduced discomfort (ASQ-Discomfort, *r* = .57), and moderately correlated with increased confidence (ASQ-Confidence, *r* = −.40) and the need for approval (ASQ-Need for approval, *r* = .39) within attachment relationships.

## Discussion

This case series aimed to assess whether an adolescent adaptation of RO DBT (RO-A) is associated with improvements in overcontrol characteristics, relationship quality and psychiatric symptoms of depression and eating disorders. The results showed that the majority of measured individual and relationship factors associated with overcontrol improved significantly with large effect size. Significant reductions in cognitive inflexibility, risk aversion and the maladaptive use of suppression of emotional expression to regulate emotions were observed, as well as improvements in reward processing. Large effect size improvements were also reported for almost all attachment and relationships factors measured. At the end of treatment adolescents were significantly less socially withdrawn, reported being more socially connected to the people around them and reported more confidence, comfort in and importance of attachment relationships.

Nevertheless, not all aspects of overcontrol measured in this case series changed during treatment. No changes were observed in levels of perfectionism, workaholism or punctiliousness; all behavioural aspects of maladaptive overcontrol. Similarly, while adolescents reported anticipating pleasant events more after RO-A, there was no change in their experience of it in the moment. Lastly, while the use of suppression of emotional expression reduced, the use of more adaptive cognitive strategies to regulate emotions remained stable. The latter finding is unsurprising in some ways, as emotional expression is a direct target of RO-A, whereas cognitive strategies are less so.

This pattern of findings suggests temperamental factors and the behavioural aspects of conscientiousness and perfectionism may not shift with RO-A treatment, despite large and clinically meaningful improvements in many other areas; namely relationships, cognitive flexibility, emotion regulation skills and mental health symptoms. This is partly at odds with evidence that perfectionism can improve with other psychological treatment [53, 54], although there is very little data for children and adolescents specifically [54].

One interpretation of this pattern of findings is that the young people are less motivated to change these facets of overcontrol. Perfectionism, workaholism and punctiliousness are all behaviours strongly reinforced within the current British cultural context and have been increasing for decades [55]. Reducing these behaviours is potentially considerably less socially and culturally acceptable than improving relationship quality, psychiatric symptom reduction and more effective emotional expression. Given these behaviours might be considered adaptive by young people, a question for future research will be whether they continue to be associated with ongoing distress or reduced functioning.

Even if some of factors associated with overcontrol do not shift in treatment, what RO-A does appear to do is provide adolescents with a range of skills to mitigate against several potentially persistent and unchanging temperamental and behavioural factors in more adaptive ways. Denissen and colleagues’ [56] model of temperament and personality development suggests temperamental factors may be managed and even slightly modified with incremental practice of self-regulation mechanisms. Perhaps the significant improvements reported in emotional regulation strategies, risk aversion, cognitive flexibility and social connection following RO-A may be the precursor to broader changes in these more persistent characteristics. Follow-up studies could help elucidate whether these unchanged factors, as well as other overcontrol factors, continue to shift after discharge. This will be an important focus of future research.

In addition to improvements in overcontrol and relationship factors, significant improvements in depressive symptoms, frequency of self-harm behaviours and eating disorder symptoms were reported following RO-A. Exploratory correlation analyses also suggest that improvements in most areas of maladaptive overcontrol are moderately to strongly associated with improvements in psychiatric symptoms of depression and eating disorders. Specifically, psychiatric symptom improvement is associated with increased relationship quality and comfort in attachment relationships, as well as aspects of reward processing and emotion regulation. The exploratory nature and design of this case series does not allow for an assessment of the nature or direction of these relationships; however, the findings offer very exploratory and preliminary evidence that targeting overcontrol in treatment is associated with psychiatric symptom improvement. Whether improvements in overcontrol mediate improvements in psychiatric symptoms, as is proposed by the RO DBT model, or vice versa, is beyond the exploratory nature of the current study but will be an important focus of future research.

One interesting finding from the current case series was the large variability in treatment dose. The number of skills classes (range = 9–34) and individual sessions (range = 8–42) varied considerably, as did the overall duration of treatment (range = 15–62 weeks). As this was a pilot test of a new model, treatment length was intentionally flexible and left to the discretion of the clinician, young person, and RO-A team consult. In some instances, clinicians and young people tended to continue individual sessions after completing a full round of skills classes, typically tapering down session frequency thereafter. Depending on need, some young people continued in the service for some time with infrequent check-in sessions to revisit skill use and monitor progress. Sometimes clinicians and young people decided to extend skills classes beyond the full dose of 20, so that the young person could strengthen newly acquired skills or end at a point that fitted with an event in the calendar year (e.g., end of a school term). A controlled trial is needed to test whether the 20-session model is sufficient for most, or whether some infrequent individual sessions post-skills class need to be built into the RO-A model.

Together these findings are encouraging and provide preliminary evidence that the 20 session RO-A model may be a new and effective way of helping a transdiagnostic group of young people struggling with multiple and significant difficulties. This transdiagnostic approach appears to lead to change in individual, social and clinical domains for adolescents with high rates of comorbidity and who have already received other treatments.

### Limitations and future directions

Despite these promising findings, there are several important limitations to this case series. The small sample size, uncontrolled design, sole reliance on self-report measures, use of adult measures in the absence of validated adolescent measures, and missing data make interpretation of the findings tentative. Several measures/subscales also had borderline acceptable internal consistency (Cronbach’s alpha < 0.7). The small sample size allowed for only very preliminary testing of the model. It limits any exploration of differences in treatment response based on presentation or diagnosis at assessment. The use of adult measures may also be contributing to the partly mixed pattern of findings. Further exploration of potential differences in the way symptoms of different psychiatric diagnoses (e.g. depression, eating disorders, anxiety disorders etc) respond to RO-A and relate to overcontrol in adolescence is needed.

Due to the exploratory nature of the study, a RCT design was not used, potentially limiting the generalisability of the findings. Larger, controlled trials that include a control group are needed to more fully test the RO-A model. Studies designed to test mediation are also needed to understand the order and process of change in overcontrol, relationship quality and symptom change during treatment.

A further limitation is that treatment adherence was not assessed in this study, due to the exploratory nature and early stages of the RO-A model. Assessing treatment adherence and whether this impacts outcome is needed in future studies. Similarly, overcontrol screening tools for adolescents did not exist at the time of data collection. Recently, the Youth Over and Undercontrol Checklist (YOU-C) [57] has been published, which is the first step to adding rigour to the screening process in future studies.

For the eating disorder sub-group in this study, no adolescent was severely underweight at commencement of RO-A and all had received some prior eating disorder treatment. As such, no conclusions can be made from the current study about the efficacy of RO-A for very underweight adolescents with no treatment history. Nevertheless, the levels of eating disorder psychopathology were high, as per baseline EDE-Q global and subscale scores, highlighting the potential role of RO-A as a treatment for young people who have persisting difficulties following some weight restoration.

Lastly, missing data at discharge may indicate that the number of measures used was not acceptable to adolescents, which needs to be considered when designing future studies. Acceptability of RO-A as a treatment more generally was not formally assessed in this case series. The low dropout (*n* = 1) and non-starter (*n* = 3) rate suggests acceptability, however more rigorous assessment is needed. Despite these important limitations, this study offers preliminary evidence that RO-A may be effective and strongly support the need for further, more rigorous testing of the model.

## Conclusion

Preliminary data from this case series suggests Radically Open Dialectical Behaviour Therapy for Adolescents (RO-A) may be an effective new transdiagnostic treatment for adolescents with maladaptive overcontrol. In this case series, RO-A supported adolescents with multiple and significant mental health difficulties to adaptively express their emotions more, increase flexibility, improve aspects of reward sensitivity, improve attachment and relationship quality alongside improvement in mood, rates of self-harm and eating disorder psychopathology. These very preliminary findings provide tentative support for RO-A as a new treatment for a group of vulnerable young people with multiple difficulties and a relatively poor prognosis. It also offers a new way of supporting adolescents with social and relationship difficulties in a targeted and defined way, an area rarely targeted directly with psychological treatments to date. RO-A also offers a new way to conceptualise recovery for adolescents struggling with one or more of the related psychiatric difficulties associated with maladaptive overcontrol and offers a new treatment direction. Nevertheless, these findings need to be replicated and tested in larger, controlled trials as this is very early, preliminary data with a small group of young people and moderate levels of missing data. Testing of the model with underweight adolescents with eating disorders is also needed.

## Data Availability

Data generated for this case series are available upon request.

## Abbreviations

%mBMI: Percentage of median body mass index
ASQ: Attachment Styles Questionnaire
CAMHS: Child and Adolescent Mental Health Service
CBT: Cognitive Behavioural Therapy
DAWBA: Development and Wellbeing Assessment
DBT: Diagnostic and Statistical Manual, 5th edition
DSM-5: Diagnostic and Statistical Manual, 5th edition
EDE: Eating Disorder Examination
EDE-Q: Eating Disorder Examination Questionnaire
ERQ: Emotion Regulation Questionnaire
FFOCI: The Five Factor Obsessive Compulsive Inventory – Short Form
FT-ED: Eating disorder focussed family therapy
ICD-10: International Classification of Diseases, 10th edition
MCCAED: Maudsley Centre for Child and Adolescent Eating Disorders
MFQ: Mood and Feelings Questionnaire
N&S: National and Specialist
OCD: Obsessive-compulsive disorder
PTSD: Post-traumatic stress disorder
RO A: Adolescent adaptation of Radically Open Dialectical Behaviour Therapy
RO DBT: Radically Open Dialectical Behaviour Therapy
SCS-R: Social Connectedness Scale - Revised
SLaM: South London and Maudsley
SNAP-Y: Schedule of Non-adaptive and Adaptive Personality for Youth
SPSS: Statistical Package for the Social Sciences
TEPS: Temporal Experience of Pleasure Scale
TEPS-ANT: Anticipation subscale of the Temporal Experience of Pleasure Scale
TEPS-CON: Consummatory subscale of the Temporal Experience of Pleasure Scale
YSR-W: Withdrawal subscale of the Youth Self-Report questionnaire
